# Design and Optimization of Hemispherical Resonators Based on PSO-BP and NSGA-II

**DOI:** 10.3390/mi14051054

**Published:** 2023-05-16

**Authors:** Jinghao Liu, Pinghua Li, Xuye Zhuang, Yunlong Sheng, Qi Qiao, Mingchen Lv, Zhongfeng Gao, Jialuo Liao

**Affiliations:** College of Mechanical Engineering, Shandong University of Technology, Zibo 255000, China; liuf162999@163.com (J.L.);

**Keywords:** PSO, BP neural network, NSGA-II, MEMS HRG

## Abstract

Although one of the poster children of high-performance MEMS (Micro Electro Mechanical Systems) gyroscopes, the MEMS hemispherical resonator gyroscope (HRG) is faced with the barrier of technical and process limits, which makes it unable to form a resonator with the best structure. How to obtain the best resonator under specific technical and process limits is a significant topic for us. In this paper, the optimization of a MEMS polysilicon hemispherical resonator, designed by patterns based on PSO-BP and NSGA-II, was introduced. Firstly, the geometric parameters that significantly contribute to the performance of the resonator were determined via a thermoelastic model and process characteristics. Variety regulation between its performance parameters and geometric characteristics was discovered preliminarily using finite element simulation under a specified range. Then, the mapping between performance parameters and structure parameters was determined and stored in the BP neural network, which was optimized via PSO. Finally, the structure parameters in a specific numerical range corresponding to the best performance were obtained via the selection, heredity, and variation of NSGAII. Additionally, it was demonstrated using commercial finite element soft analysis that the output of the NSGAII, which corresponded to the Q factor of 42,454 and frequency difference of 8539, was a better structure for the resonator (generated by polysilicon under this process within a selected range) than the original. Instead of experimental processing, this study provides an effective and economical alternative for the design and optimization of high-performance HRGs under specific technical and process limits.

## 1. Introduction

The MEMS hemispherical resonator gyroscope (HRG) is one of the typical representatives of micro-resonator gyroscopes for its high reliability, good accuracy, strong stability, long service life, and simple structure [1,2]. The existing MEMS hemispherical resonator gyroscopes have achieved bias stability and a Q factor to the level of 0.01°/h and 1,000,000, respectively. To adapt it to a different application scenario, researchers have developed the micro HRG for different materials and structures, and for these different gyros, there are a few discrepancies in their manufacturing processes.

Up until now, there have been three main approaches to processing a MEMS hemispherical resonator. Since the approaches are different, the resonators have different modeling characteristics. Additionally, since the manufacturing processes are different, the resonators have different modeling characteristics [3].

Andrei M. Shkel et al. from the University of California proposed the method of using glass blow molding to make the resonator of a MEMS HRG [4]. K. Najafi et al. from the University of Michigan developed a MEMS HRG with a bias stability of 0.0391°/h and an angular random walk of 0.0087°/√ h by applying a constant velocity of airflow directly above the fused silica [5]. 

On the other hand, it is difficult to control the concentricity error and roundness error of a resonator processed via this method, as Shanghai Jiao Tong University demonstrated [6].

Based on the silicon substrate, our research group obtained a hemispherical cavity via wet etching and then matched it with a hemispherical shell resonator using a polysilicon thin film deposition process [7]. J. M. Gray et al. from the University of Colorado in the United States obtained a resonator, of which the thickness was as small as 50 nm based on ALD (atomic layer deposition) [8]. However, the dimensional parameters of a resonator processed through this method usually stay at an uncontrollably larger size, as the National University of Defense Technology has shown [9,10].

The Shanghai Institute of Optics and Fine Mechanics, the Chinese Academy of Sciences, and the National University of Defense Technology all developed a resonator successfully by laser etching, whose diameter was 39 nm and 1 mm, respectively [11,12].

Although there has been a flood of research to reveal the manufacturing process mechanisms to improve the modeling quality of the resonator, many problems remain [11,13], as shown in Table 1. In contrast to the macroscopic hemispherical gyroscope, whose shaping characteristics can be eliminated via accurate post-processing, the MEMS hemispherical gyroscope has obvious shaping characteristics in its forming structure that cannot be compensated for due to its excessively small size. It is known that a different gyro structure corresponds to a different processing method and that this cannot be chosen [14,15]. So, experience always plays an important role in the design of a resonator, which cannot be separated from experimental exhaustion and processing. On the one hand, it is inefficient to obtain all of the resonator structures through enumeration; on the other hand, it is impossible to obtain the optimal structure through enumeration without consideration for the characteristics corresponding to the existing manufacturing process. Thus, the question of how to obtain the optimal structure under the restriction of a specific manufacturing process using a systematic approach is one of the most important projects for micro-HRG.

## 2. Data Collection and Analysis Based on PSO-BP and NSGA-II

**Figure 1:**
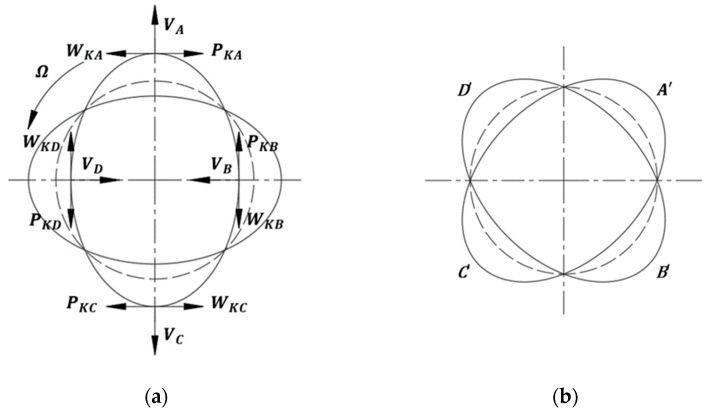
Operating mechanism of HRG: (**a**) drive mode of the HRG and (**b**) detect mode of the HRG.

As Figure 1 shows, MEMS HRG achieves its high-precision detection through the standing wave influenced by Coriolis affecting the resonator. The incentive part affects periodic excitation caused by coulomb forces or mechanical forces to the resonator; then, a standing wave will come into play on the resonator. The antinode points of the standing wave are set as A, B, C, and D; when the shell resonator is stimulated by an angular velocity Ω relative to the inertial system, each antinode point will have the implicated angular velocity Ω. 

Under the effect of V_A_, V_B_, V_C_, and V_D_ caused by the standing wave and the implicated angular velocity Ω external input of A, B, C, and D have Coriolis accelerations of W_KA_, W_KB_, W_KC_, and W_KD_, respectively. Thus, the Coriolis inertial force of P_KA_, P_KB_, P_KC_, and P_KD_ are generated by the Coriolis accelerations of V_A_, V_B_, V_C_, and V_D_. Two equivalent Coriolis couples with opposite directions are formed via P_KA_–P_KC_ and P_KB_–P_KD_, resulting in the precession of the standing wave relative to the resonator shell and inertial reference frame. 

Due to the precession of the standing wave, the points on the harmonic oscillator that have an isometric deformation will change to other ones. Measuring electrical signal changes at specific points caused by their deformation changes, the accurate angle and angular velocity of the external input will be determined.

The signal quality is affected by multiple factors, of which the most significant effect is the Q factor and frequency characteristic. 

The Q factor represents the ratio of the total energy that the resonator stored to the energy that dissipated in a vibration cycle. There are a few elements that influence the Q factor, such as thermoelastic damping, anchor damping, surface damping, and air damping. The Q factor can be written as follows [16]:

(1) $$\frac{1}{Q}=\frac{1}{Q_{\mathrm{surface}}}+\frac{1}{Q_{\mathrm{ted}}}+\frac{1}{Q_{\mathrm{air}}}+\frac{1}{Q_{\mathrm{anchor}}}$$

Air damping represents the energy consumption caused by air viscous force to a certain degree. However, since the MEMS resonator always works in a vacuum environment, it does not work obviously in energy consumption for the air damping.

The anchor damping represents the energy consumption caused by anchor loss. When the resonator vibrates, a part of the energy will be lost through the basement to the earth, and that is what is called anchor loss.

Surface damping represents the energy consumption caused by surface defects such as large surface harshness. A rough surface causes increasing surface quality imbalances and other problems, which make the resonator have to carve out a portion of energy to keep balance.

Thermal elastic damping represents the energy consumption caused by the irreversible heat flow. Under the effect of the standing wave, the shell resonator receives tension and compression at the different parts; then, the temperature of the different parts under tension and compression decreases and increases, respectively, resulting in a local temperature difference in the resonator.

For the MEMS HRG, the size and deformation of the anchor are too small to make the anchor loss give considerable influence on the Q factor compared with the thermal electric damping. Additionally, as shown above, surface damping and air damping can also be ignored. So, what we have to take into consideration is only thermal elastic damping.

The frequency characteristic includes characteristic frequency and frequency cracking. Characteristic frequency is the natural frequency of a specific vibration mode; it is influenced by the structure and materials of the resonator. Frequency cracking is the difference between the operating mode and its parasitic mode; it is influenced by structure mass imbalance and stiffness imbalance of the resonator. 

The operating mode of HRG is the natural-bending vibration mode, also known as the four-antinode mode. Generally, the ambient noise is around 5000 Hz, aiming at minimizing the disturbance to measurement signal caused by ambient noise; the characteristic frequency of operating mode should keep a distance to the frequency of 5000 Hz.

### 2.1. Preparation

For this study, an ideal model was used, in which the stiffness and mass are absolutely symmetrical; thus, the frequency cracking was not taken into consideration.

Additionally, for the resonator to have other modes at low-frequency ranges except for the operating mode, it is necessary to make the difference between the operating mode and other low-frequency modes as large as possible.

**Figure 2:**
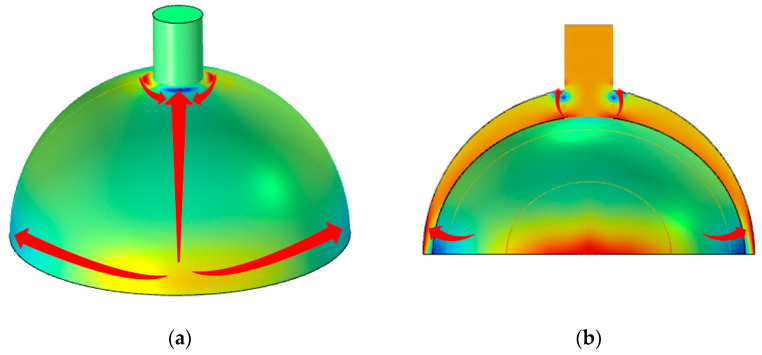
The trajectories of irreversible heat flow in operating mode: (**a**) heat flow on the surface and (**b**) heat flow on the cross-section.

To optimize the resonator based on the analysis of thermal elastic damping, the trajectories of the irreversible heat flow should be determined. In the heat conduction of the solid module and solid mechanics module of COMSOL, as the finite element simulation results revealed, the trajectories of the irreversible heat flows are shown in Figure 2.

There are five tracks for the irreversible heat flows (refer to [17]). The heat flows along the lip of the resonator and the anchor of the hemispherical shell surface are relatively long, and the heat transfer time is much longer than the vibration period of the operating mode. Therefore, the energy loss caused by these three heat flows can be ignored. Then, the thermo elastic damping of the resonator can be written as follows:

(2) $$\frac{1}{Q_{\mathrm{TED}}}=\frac{1}{Q_{R}}+\frac{1}{Q_{r}}$$

(3) $$Q_{R}=(\frac{E\alpha^{2}T_{0}}{C\rho })\frac{\omega \cdot \tau_{\mathrm{across}-t}}{1+({\omega \cdot \tau_{\mathrm{across}-t})}^{2}}$$

(4) $$\frac{1}{Q_{r}}=(\frac{C\rho }{E\alpha^{2}T_{0}})\frac{1+({\omega \cdot \tau_{\mathrm{across}-z})}^{2}}{\omega \cdot \tau_{\mathrm{across}-z}}+(\frac{C\rho }{E\alpha^{2}T_{0}})\frac{1+({\omega \cdot \tau_{\mathrm{across}-s})}^{2}}{\omega \cdot \tau_{\mathrm{across}-s}}$$

(5) $$\tau_{\mathrm{across}-t\;}=\frac{t^{2}\rho C}{\pi^{2}k}$$

(6) $$\tau_{\mathrm{across}-z}=\frac{{\overset{-}{z}}^{2}\rho C}{\pi^{2}k}$$

(7) $$\tau_{\mathrm{across}-s}=\frac{s^{2}\rho C}{4k}$$

ρ, C, E, α, and T_0_ are the density, constant pressure heat capacity, Young’s modulus, coefficient of thermal expansion, and initial temperature of the harmonic oscillator material, respectively; τ_across-h_, τ_across-z_, and τ_across-s_ are time constants for the heat flow transfer through the thickness direction of the hemispherical shell, the heat flow transfer through the thickness direction at the anchor, and the heat flow along the circumferential direction of the edge of the anchor, respectively; t is the thickness of the edge of the shell; $\overset{-}{z}$ is the average length of the heat flow passing through the thickness direction at the anchor; s is the radius of the hemispherical shell at the edge of the anchor.

**Figure 3:**
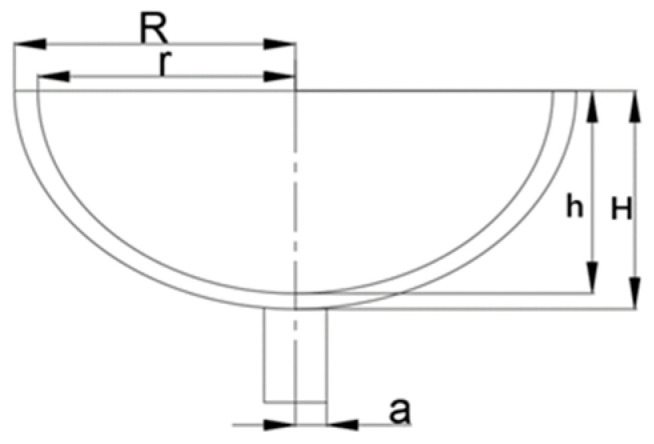
The structure-parameters of resonator.

It can be inferred that structure parameters that have an influence on the thermo elastic damping are t, $\overset{-}{z}$, and s from (5)–(7). Additionally, the fundamental parameters of the structure parameters are a, R, r, H, H − h, and R − r (refer to Figure 3).

**Figure 4:**
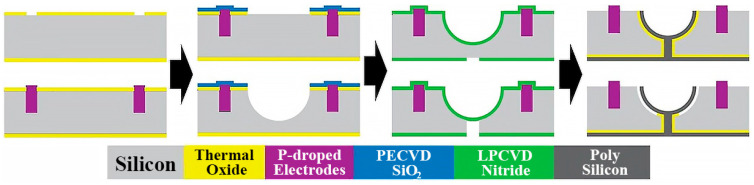
Process for preparation of resonator by thin film deposition.

Additionally, for this study, a polysilicon resonator processed via thin film deposition was taken as an example. The process of thin film deposition can be obtained from [18], as shown in Figure 4. First, the thermal oxide is patterned to mask the wafer, capacitive actuation, and sense electrodes that are isolated from the substrate are created by PN junctions. Second, A PECVD oxide layer is patterned to form the isotropic etch mask, and sulfur hexafluoride (SF6) plasma is used to etch the hemispherical mold. Third, an LPCVD low-stress silicon nitride layer is patterned to mask the topside and backside during KOH etching, which forms the back-side plug mold. Finally, the polysilicon layer on the wafer surface is removed in a short dry etching step with SF6 plasma while the shell is protected by a photoresist, and the shell is then released in HF.

There are mainly four steps for thin film deposition to process a resonator: first, manufacturing a mask on the silicon substrate; second, etching the silicon substrate to form a mold; third, depositing the structure in the mold; fourth, releasing the structure via wet etching.

The forming defects of the resonator processed via this method are mainly caused by the mold, which may cause a rough surface and a cavity structure, with the ratio of depth to width <1 resulting from wet etching, which cannot be resolved faultlessly now. 

As mentioned earlier, the forming defects of specific manufacturing processes were the problems that should be put first. So, in consideration of the modeling characteristic and gravity effect when forming the structure via deposit, the parameter characteristics of the resonator can be written as follows:

(8) AS=b

(9) $$A=\left[\begin{array}{ccccc} 1 & -1 & 0 & 0 & 0\\ -1 & 1 & 0 & 0 & 0\\ 1 & 0 & 0 & -1 & 0\\ -1 & 0 & 0 & 1 & 0 \end{array}\right]$$

(10) $$S=\left[\begin{array}{ccccc} R & r & H & h & a \end{array}\right]$$

(11) $$b\;=\left[\begin{array}{c} 2\\ 0.5\\ 50\\ 50 \end{array}\right]$$

**A** is the coefficient matrix; **S** is the structure parameters matrix; **b** is the deviation matrix.

Set t as follows:

(12) t=H−h

Referring to [18], the varying range of structure parameters was set as shown in Table 2, and polysilicon was selected to deposit the structure.

Obtaining the mapping between structure parameters and performance parameters requires a lot of calculations; finite element simulation can solve the problem of data collection, and BP neural networks can solve the problem of fitting efficiently.

The variation is regular between performance parameters, and singular structure parameters can be obtained from simulation by varying one of the structure parameters and holding on to the others sequentially. Some of the results are shown in Table 3.

**Figure 5:**
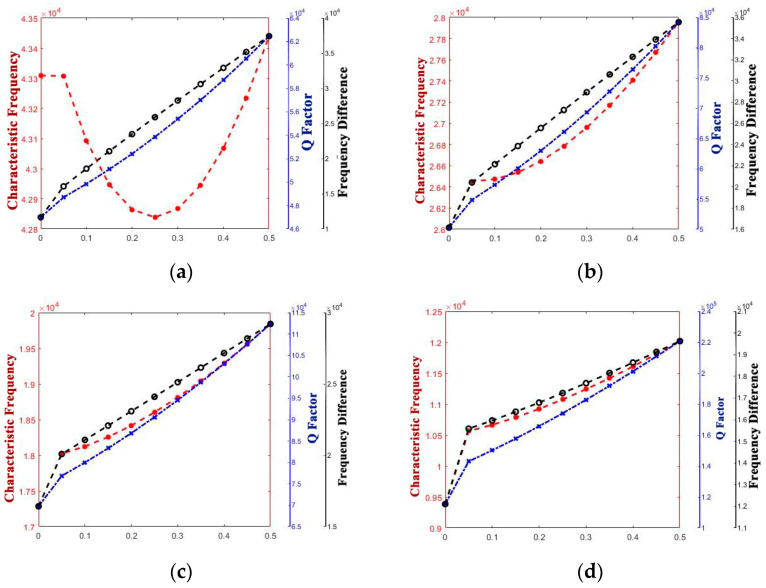
Variety regulation between R and performance parameters. (**a**) r = 300; (**b**) r = 400; (**c**) r = 450; (**d**) r = 500.

**Figure 6:**
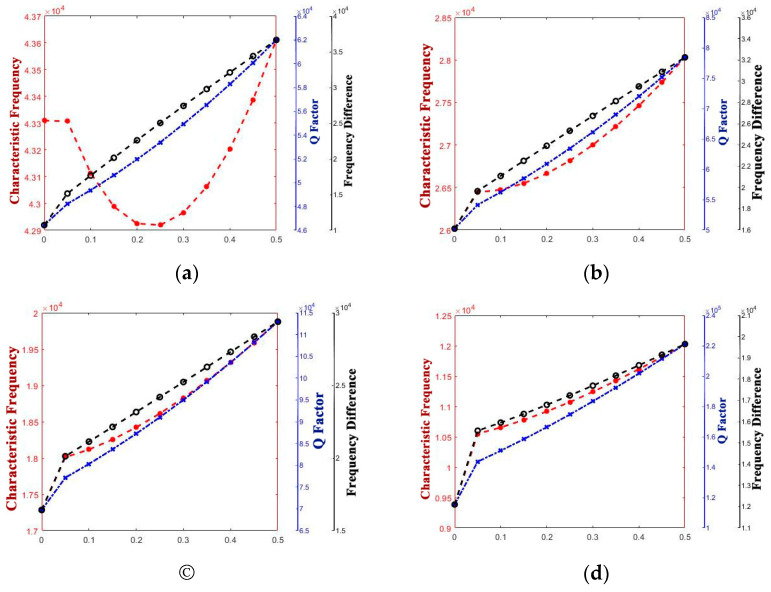
Variety regulation between r and performance-parameters (**a**) R = 300; (**b**) R = 400; (**c**) R = 450; (**d**) R = 500.

**Figure 7:**
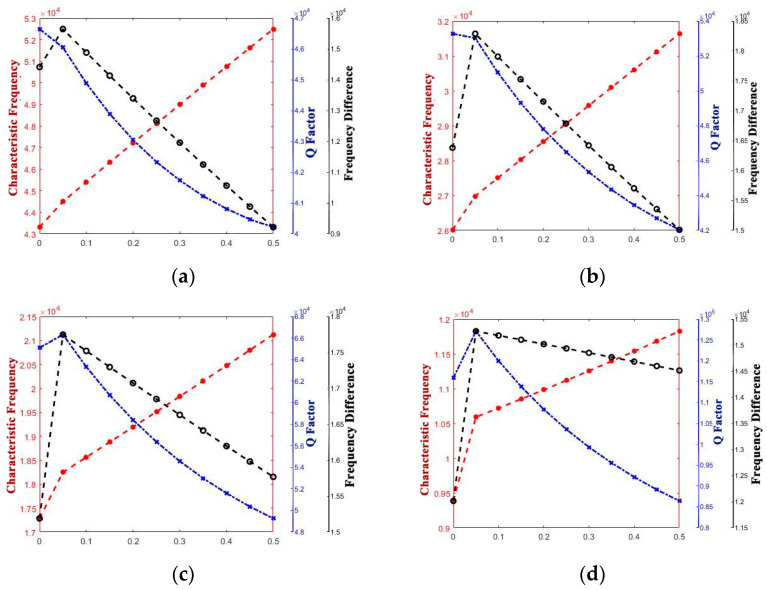
Variety regulation between t and performance-parameters (**a**) R = 300; (**b**) R = 400; (**c**) R = 450; (**d**) R = 500.

**Figure 8:**
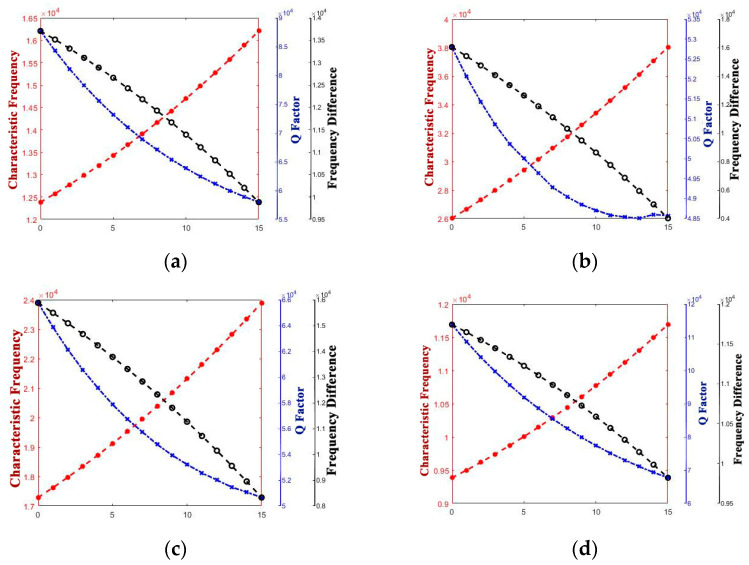
Variety regulation between a and performance parameters. (**a**) R = 300; (**b**) R = 400; (**c**) R = 450; (**d**) R = 500.

**Figure 9:**
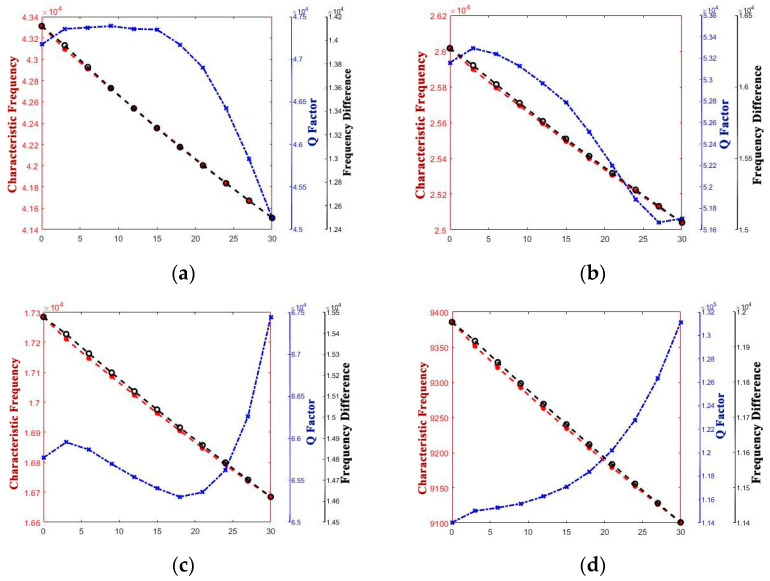
Variety regulation between h (increase) and performance parameters (**a**) R = 300; (**b**) R = 400; (**c**) R = 450; (**d**) R = 500.

**Figure 10:**
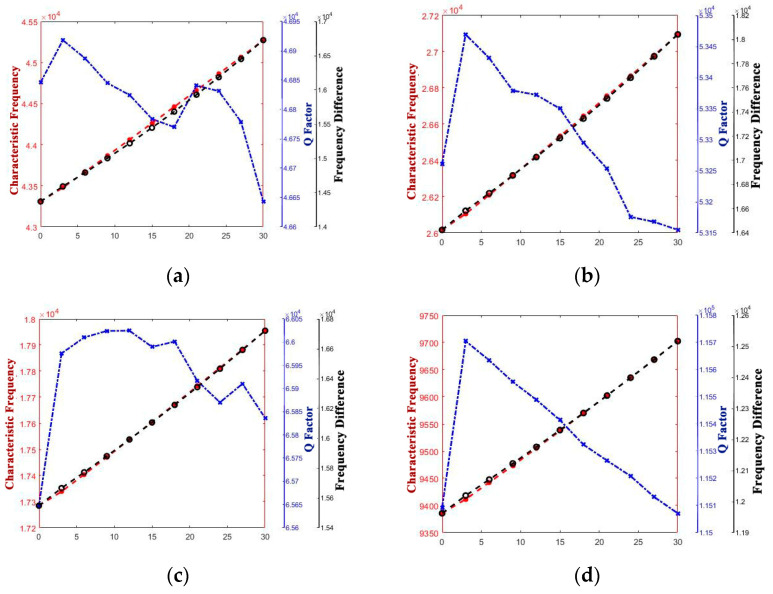
Variety regulation between h (decrease) and performance parameters. (**a**) R = 300; (**b**) R = 400; (**c**) R = 450; (**d**) R = 500.

The curve graphs drawing the singular structure parameter with all of the performance parameters are shown in Figure 5, Figure 6, Figure 7, Figure 8, Figure 9 and Figure 10. For every structure parameter, the figure (a–d) demonstrate when R or r is 300, 350, 400, and 500 as samples.

It can be seen that the effects of the performance parameters of varying R and r are almost the same, and it is in conformity with the conclusion that [17] provided. The increase in R and decrease in r both result in the increase in thickness of the edge of the shell, and it affects the path length of the heat flow transfer through the thickness direction of the hemispherical shell and the heat flow transfer through the thickness direction at the anchor. 

Increasing the thickness of the hemispherical shell appropriately can extend the path length of the heat flow transfer through the thickness direction of the hemispherical shell and the heat flow transfer through the thickness direction at the anchor, decreasing the energy loss in a vibration period, which results in the decrease in thermoelastic damping, improve the Q factor, and raise the characteristic frequency. 

However, whether it is the Q factor, the characteristic frequency, or the frequency difference, there is a turning point when the structure transfers from the standard hemisphere to the non-standard hemisphere. It can be inferred that it is the sudden change in the structure that results in the sudden change in the path length for the irreversible heat flow. 

It is obvious that except for the turning point, there is a significant linear positive correlation between t and frequency difference and a linear negative correlation between t and characteristic frequency. It means that the characteristic frequency of the operating mode is getting closer and closer to the characteristic frequency of the other mode, which can also be said that the characteristic frequency is getting more and more intense. So, it is necessary for us to make the value of t as small as possible.

Additionally, for the Q factor, it is also necessary to control the size of t at a low level as it shows a negative correlation between t and the Q factor since the heat production converged from the edge to the bottom, which results in the increase in energy loss with the t increase.

It can be informed that the characteristic frequency increases as a increases. However, the Q factor and frequency difference are both generally negatively correlated with a; it can be inferred that although the path length of the heat flow at the anchor increased, the heat flow generated at the bottom also increased while a increased.

Figure 9 and Figure 10 show the variety of regulation between performance parameters and h. 

When H and h increase synchronously, out of our expectation, the Q factor decreases within a certain range when R is less than 500 μm, and then the Q factor increases, which is par for the course. An inference that the Q factor decrease because of the increase in energy loss caused by heat flow along the circumferential direction of the edge of the anchor is less than the decrease in energy loss caused by heat production converging from the edge to the bottom was given. Additionally, this phenomenon disappears when R is large enough. 

When H and h decrease synchronously, predictably, the Q factor decreases as the energy loss caused by heat flow along the circumferential direction of the edge of the anchor decreases. Since the path length of heat flow along the circumferential direction of the edge of the anchor is reduced, this results in a reduced transfer time.

It can be seen that although the curve graphs can reflect the relationship between single structure parameter and performance parameters to some extent, it is difficult to explain the mapping reasonably since every structure parameters interact with each other, and it is, hence, the need for global optimization of all of the structure parameters.

### 2.2. Data Handling

**Figure 11:**
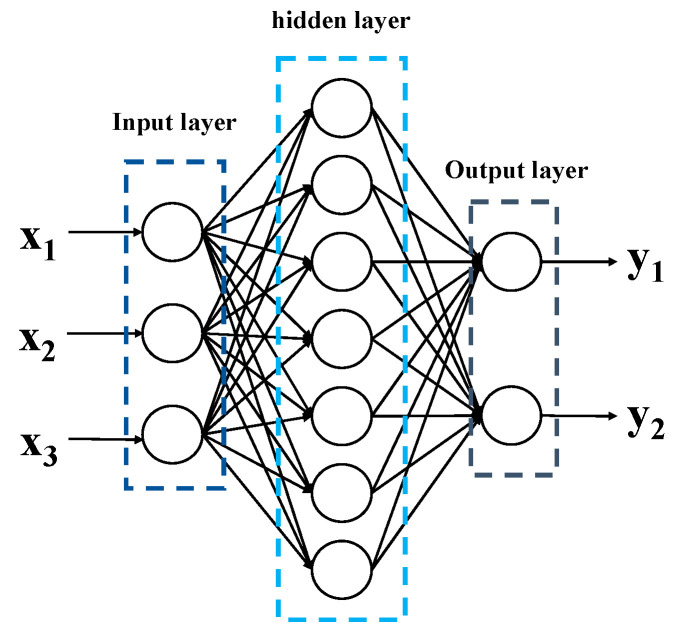
The structure of BP neural network.

BP neural network is one of the most widely used neural network models as a multi-layer feedforward neural network whose training strategy is according to an error backpropagation algorithm, and its structure is shown in Figure 11. We can obtain the mapping between multiple arguments and multiple variables via the BP neural network.

However, the adjusted direction of weight values of traditional BP neural network is along the direction of local improvement, which leads to local extremum easily. Moreover, due to the BP neural networks being sensitive to initial weight values, different initial weight values may lead the neural network convergence to different local minima; in addition, there lacks an effective selection method to determine the weight values, which can only be obtained through experimental learning and training, while too many hidden layer neurons may lead to over learning, but too few causes under learning. Additionally, another problem is that the number of hidden layer neurons can only be roughly obtained based on experience, making the algorithm unstable.

**Figure 12:**
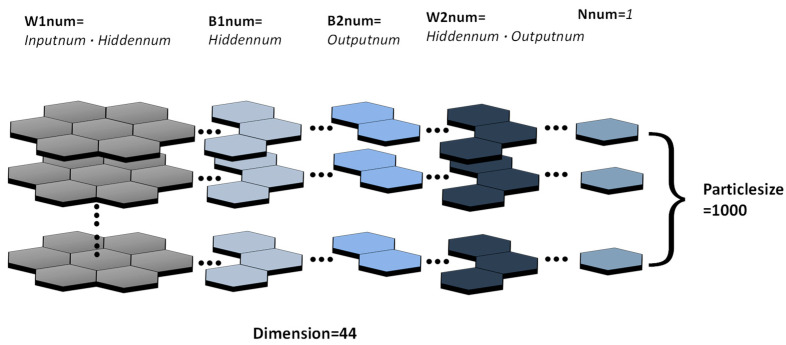
The structure of a particle for PSO-BP.

Particle Swarm Optimization (PSO) can solve the above problems of traditional BP neural networks to some extent. We assume that the deviations, weight values, and a number of hidden layer neurons of the neural network as a disaggregation set to be solved, and all of the disaggregation sets are treated as a population in the PSO, while each disaggregation set is treated as an individual, as shown as Figure 12. This way, we can prevent the optimal weight values and deviation obtained from falling into the locally optimal solution by combining the locally optimal solution (individual optimal solution) and the globally optimal solution (population optimal solution) and obtain uniquely determined weight values, deviation, and a number of hidden layer neurons.

N is the number of hidden layers neurons; W1num, B1num, W2num, B2num, and N num correspond to the dimensions of W1, B1, W2, B2, and N. Input num is the dimension of input data, hidden num is the dimension of hidden layer neurons, output num is the dimension of output data, and N num is 1, which is used to record the number of hidden layer neurons.

NSGA-II is a kind of genetic algorithm which is always used to solve the combinatorial optimization problem; it can improve the probability of excellent individuals being preserved and reduce global complexity with an elitist strategy and the concept of congestion degree.

**Figure 13:**
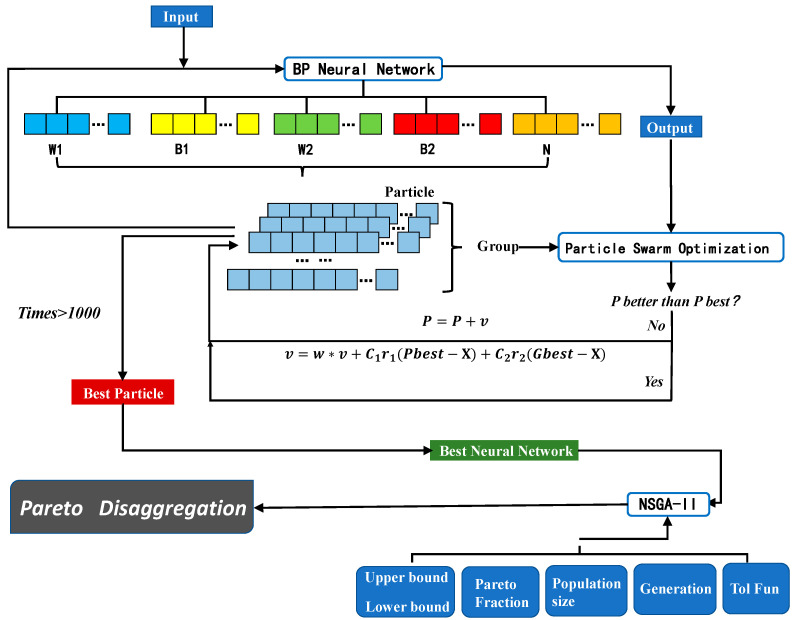
The structure of the algorithm for parameters optimization.

The mapping of structure parameters and performance parameters can be obtained via the BP neural network, and the BP neural network will be set as the constraint function of NSGA-II. Then, the best disaggregation set for the combination of structure parameters that correspond to the best performance parameters, under the limitation of a specific manufacturing processing, will come out as the output of NSGA-II, as shown in Figure 13.

The specific steps are as follows:

1. Train BP neural network.
  (1) Build a sample set from simulation data. The dimension of the input is 5, and each input contains R, r, H, h, and a. The dimension of the output is 3, and each output contains f, f_0_, and Q. There are 1760 sets of data.
    The matrix of input is as follows:
    (13) $$X={\left[R\;r\;H\;h\;a\right]}^{T}$$
    The matrix of input is as follows:
    (14) $$Y={\left[f\;f^{0}\;Q\right]}^{T}$$
  (2) Normalize the dataset as follows:
    (15) $$X_{i}=\frac{x_{i}-x_{\min}}{x_{\max}-x_{\min}}(i=1,2\ldots \ldots 1760)$$
    X_i_ is the normalized data of the No. i group, and x_i_ is the original data of the No. i group in the sample set. x_max_ and x_min_ are the original maximum and minimum in the sample set, respectively.
  (3) Optimize the initial weight values, deviation, and number of hidden neurons of the BP neural network via PSO.
    The number of particles is set to 200, and the encoding of a single particle is as follows:
    (16) $$P1_{\;}=[w_{1,1}^{1},\;\ldots \ldots ,\;w_{N,5}^{1},\;b_{1}^{1},\ldots \ldots ,\;b_{N}^{1},\;w_{1,1}^{2},\;\ldots \ldots ,\;w_{3,N}^{2},\;b_{1}^{2},\;b_{2}^{2},\;b_{3}^{2},\;N]$$
    $w_{1,1}^{1}$, ……, $w_{N,5}^{1}$ are the weight values from the input layer to the hidden layer, recorded as **W_1_**;
    $b_{1}^{1}$, ……, $b_{N}^{1}$ are the deviations from the input layer to the hidden layer, recorded as **B_1_**;
    $w_{1,1}^{2}$, ……, $w_{3,N}^{2}$ are the weight values from the hidden layer to the output layer, recorded as **W_2_**;
    $b_{1}^{2}$, $b_{2}^{2}$, $b_{3}^{2}$ are the deviations from the hidden layer to the output layer, recorded as **B_2_**;
    **N** is the number of neurons in the hidden layer.
  (4) Optimize the particles.

Giving the weight values, deviations, and the number of hidden layer neurons to the neural network every time, the particles update and import the structure parameters into the neural network. Take the two-norm difference between the predicted data and the actual data output as the evaluation criterion for every particle. The smaller the value, the closer the particle is to the optimal solution. 

**Figure 14:**
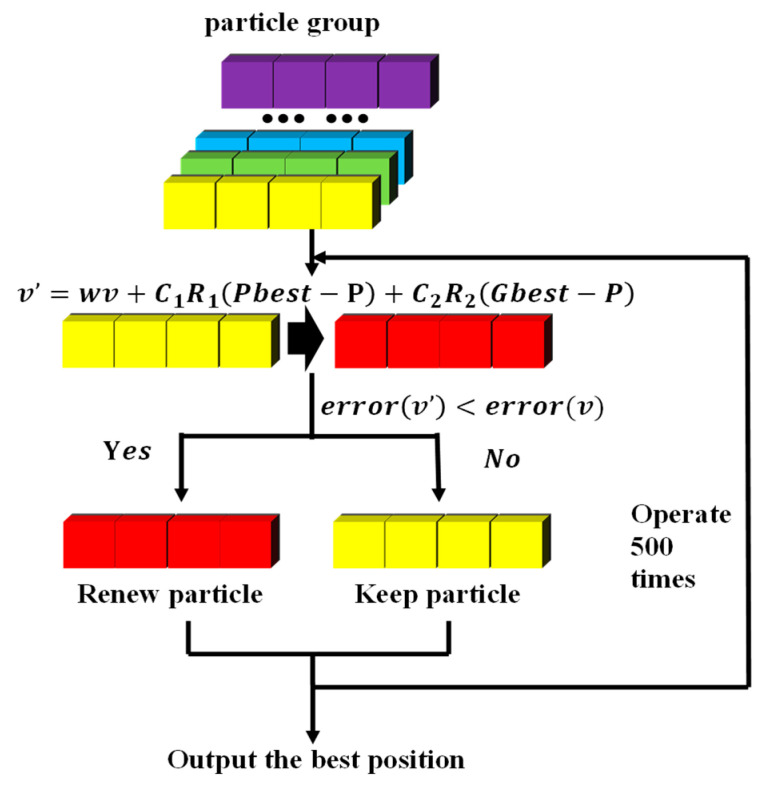
Process of particle optimization.

During this process, the current optimal position P of a single particle, which has the smallest evaluation value for a single particle, and the current optimal position G of the entire particle swarm, which has the smallest evaluation value over the whole group are recorded, and the particle position is continuously updated with the target of optimal position. The overall process is shown in Figure 14.

The function of velocity update is as follows:

(17) $$v=w\;\times \;v+C_{1}r_{1}\left(\mathrm{Pbest}-X\right)+C_{2}r_{2}\left(\mathrm{Gbest}-X\right)$$

The function of position update is as follows:

(18) P=P+v

where w is the inertia factor, C_1,_ and C_2_ are individual learning factors and social learning factors, respectively, and the value range of r_1_ and r_2_ is (0, 1).

After iterative calculation, the particle with the lowest fitness is output, and the weight values, deviation, and a number of hidden layer neurons stored within the particle are just the optimal ones for the BP neural network.

2. Calculate the best combination of structure parameters.

**Figure 15:**
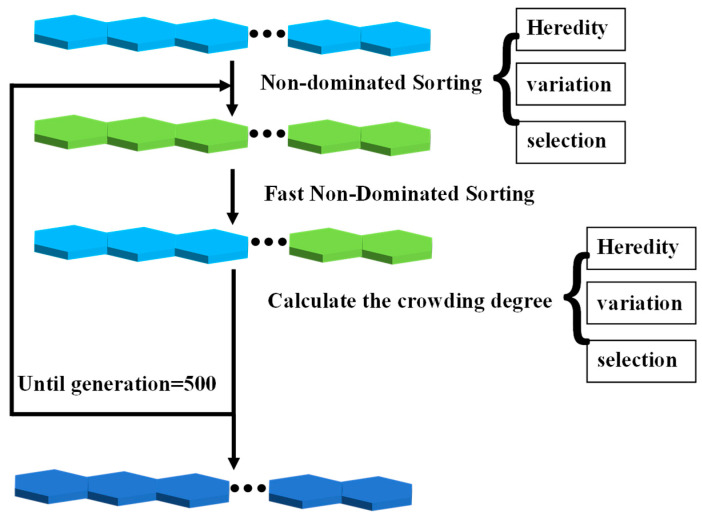
The pross of NSGA-II.

The entire process is shown in Figure 15.

(1) Pick up the information of the particle the output from 1. Give the best weight values, deviation, and a number of hidden layer neurons to the BP neural network; it will be the constraint function of NSGA-II that will be used in (2).
(2) Set the BP neural network as the constraint function of NSGA-II, and set AS=b as a constraint condition.
(3) Selecting the structure-parameter combinations that participate in genetic variation by elite strategies; Then, cod the structure-parameter combinations selected to simulate binary crossover and polynomial mutation, and put the offspring and the parent of structure-parameter combinations together. The combined structural parameter combinations were sorted via a fast non-dominant sorting algorithm, and a new parent is selected.

**Figure 16:**
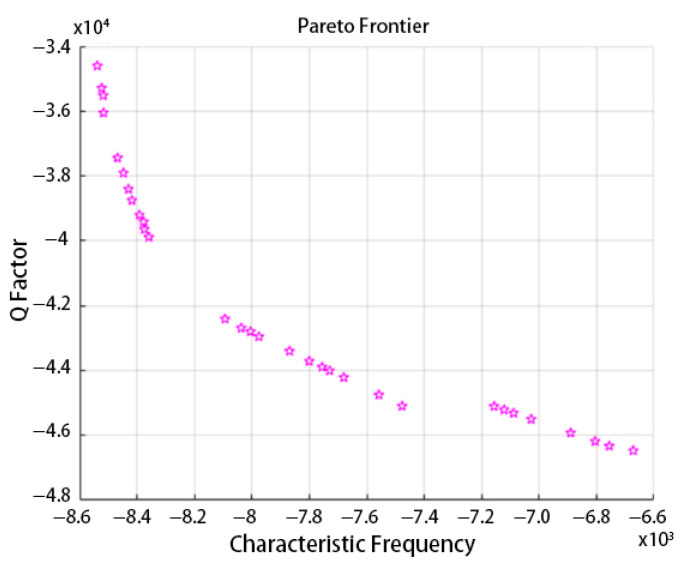
The Pareto frontier.

Repeat these 500 times, and output the Pareto frontier. The Pareto frontier is shown in Figure 16.

## 3. Results

We need a higher Q factor, a higher frequency difference between the frequency of ambient noise and the characteristic frequency of the operating mode, and a higher frequency difference between the characteristic frequency of other low-frequency modes and the characteristic frequency of the operating mode.

According to Figure 16, the Pareto frontier can be divided into four groups: the first is from point 1 to point 4; the second is from point 5 to point 12; the third is from point 13 to point 23; the fourth is from point 24 to point 31. Points in group 1 have the highest Q factor and the lowest frequency difference, and, among these points, the difference in the Q factor is unusually small, while the difference in frequency difference is relatively large. 

The difference in the Q factor among the points of the second group is second only to the first group; however, the difference in the Q factor among the points of this group is obviously larger than it of the first group, while the differences in frequency difference are almost the same.

For the third group and the fourth group, differences in the Q factor and frequency difference among them are less distinctive. However, compared with the first group and the second group, they have a considerable variation in the Q factor and frequency difference over their points inside. Additionally, the Q factor of the points in the third group and fourth group are all far below the points in the first group and second group.

As analyzed above, point 12, the last point in the second group, is selected as the best combination of the structure parameters, which corresponds to the best performance parameters. It has a Q factor of 42,382 and a frequency difference of 8323. Additionally, its structure parameters are R = 408.726, r = 406.855, t_0_ = 0.712, h = 365.505, a = 54.415.

Taking the structure parameters of the 12th point into finite element simulation, it came that the Q factor is 42,454, and the frequency difference is 8539, which is very similar to the result from PSO-BP and NSGA-II; thus, this method is proved to be available.

### 3.1. Local Analysis

Local analysis is operated to ensure that point 12 does not fall into a locally optimal solution.

Some of the structure parameters and the performance parameters corresponding to them around point 12 are listed in Table 4.

**Figure 17:**
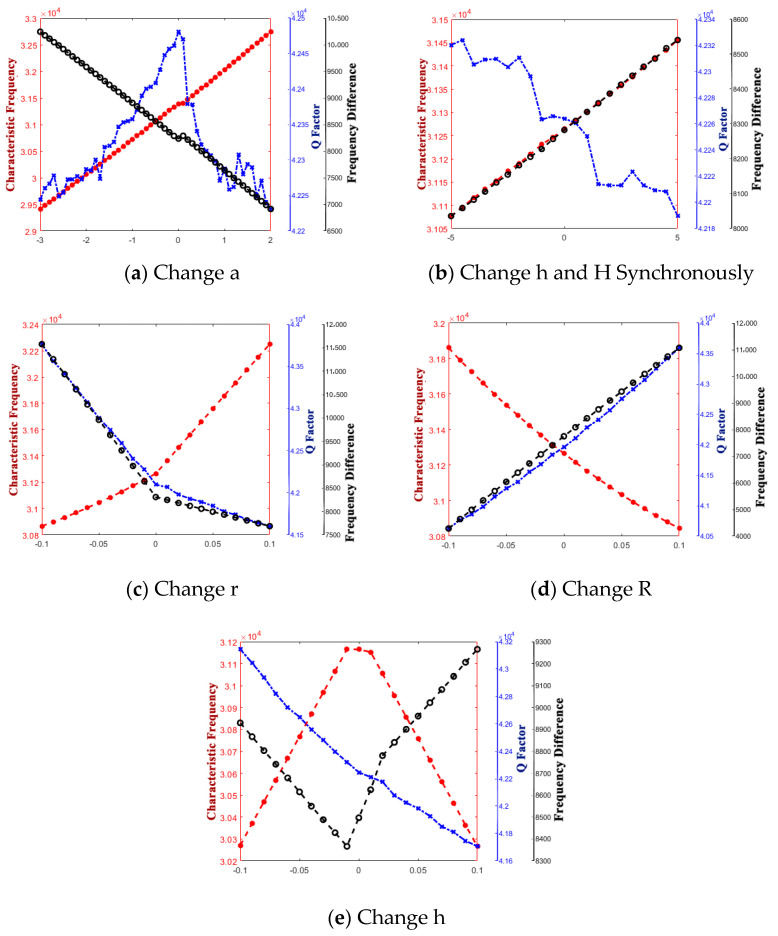
Variation of performance parameters at the local range around point 12.

Additionally, the variation of performance parameters at this local range is shown in Figure 17.

It is obvious that whichever structure parameter is changed, point 12 is located in the crossover point and knee point. When a structure parameter is changed, if point 12 did not own the highest Q factor, it is inevitable that it owns the highest frequency difference. So, point 12 is the best solution to obtain the best performance parameters at the local range.

### 3.2. Global Analysis

A global analysis is operated to ensure that point 12 is the best solution actually.

The structure parameters and the performance parameters corresponding to them in the selected value range are listed in Table 5.

**Figure 18:**
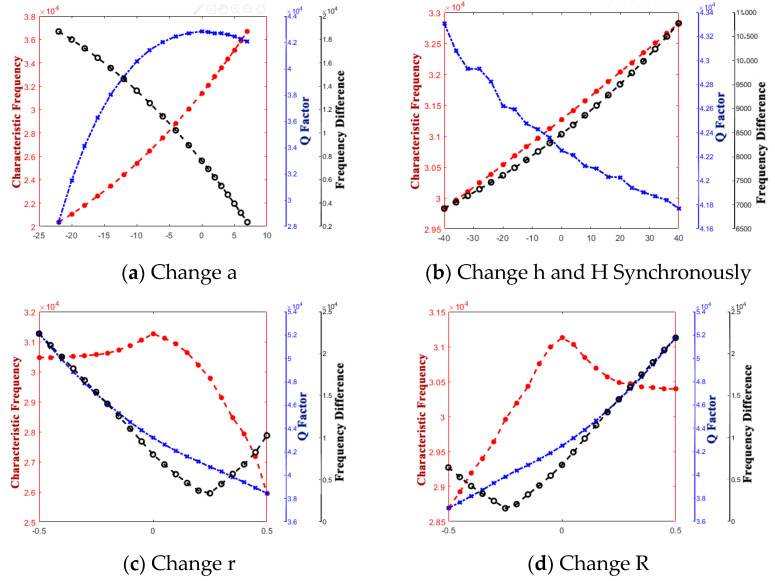

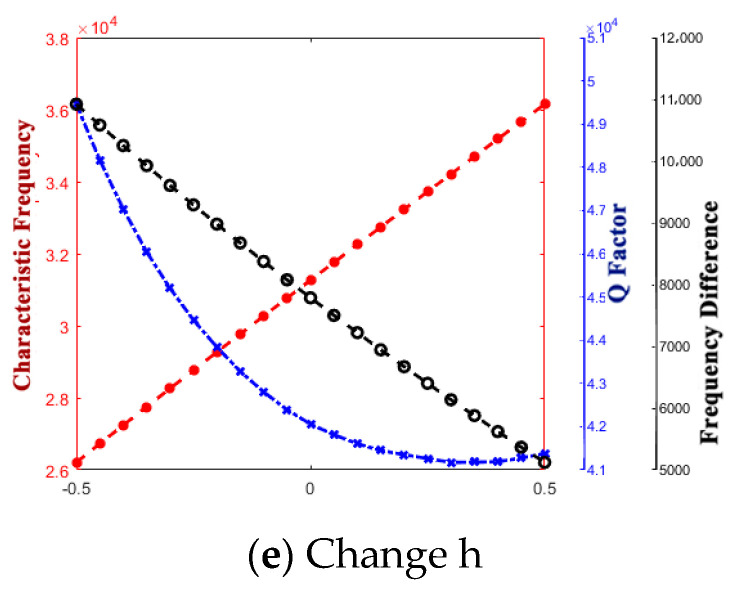
Variation of performance parameters at the global range around point 12.

Additionally, the variation of performance parameters at the global range is shown in Figure 18.

It is obvious that whichever structure parameter is changed, point 12 is located in the crossover point and knee point. When a structure parameter changes, if point 12 did not own the highest Q factor, it is inevitable that it has the highest frequency difference. So, point 12 is the best solution to obtain the best performance parameters at the global range. It has a Q factor of 42,454 and a frequency difference of 8539. Additionally, its structure-parameters are R = 408.726, r = 406.855, t_0_ = 0.712, h = 365.505, and a = 54.415. So, when the thin film deposition method is operated, the best structure has the combination of structure parameters as mentioned above.

A comparison between the simulation results of this study and experimental data from the relevant literature is as Table 6 shows. 

As Table 6 shows, the structure parameters of the polysilicon resonator given by this study have the highest Q factor among the resonators presented in reference [7,19], which is up to 42,454. The difference between the characteristic frequency of the operating mode and the frequency of the spurious mode of this resonator is 8539, ensuring that the frequency coupling between the operating mode and spurious mode is very small, which makes the influence on the hemispherical resonator generated by the spurious mode, and can be reduced to a lower level. Compared to the hemispherical resonator presented in reference [19], the radius and the thickness of the shell have a slight difference with these two parameters of the hemispherical resonator in this study. However, there is a major gap in the Q factor. Besides the differences in the material properties coming from simulation and experiment, the influence that the variety regulation mentioned above, between the structure parameters and the performance parameters, plays an important role. According to the analysis of Figure 5b, as the thickness of the shell increases, the characteristic frequency of the operating mode and the Q factor both increase continually because of the lengthening of the delivery time of heat flow, which results in the decrease in energy loss in a vibration period. 

According to the analysis of Figure 9b, there is a decrease in the Q factor when the depth of the shell increases, while the characteristic frequency of the operating mode has a different trend.

There might be a disparity in the depth of the shell between reference [7] and this study. Additionally, for reference [19], according to the analysis of Figure 5 and Figure 10, when the radius of the resonator is up to 650 μm, different from the variation trend of the characteristic frequency of operating mode, there is a decrease in Q factor when the thickness and depth of the resonator decrease, since a decrease occurs in the delivery time of heat flow, which is the cause for the increase in energy loss in a vibration period.

Since Table 6 shows that the performance parameters of the hemispherical resonator obtained via the method referring to this study are better than those of the hemispherical resonators presented in references [7,19], the feasibility that this method can be applied to the design and optimization under the limitation of a specific manufacturing process was proven.

## 4. Conclusions

As analyzed in Section 3, whether it is a local range or a global range, point 12 is the best solution. At the numerical intervals that we have set, the best combination of structure parameters is obtained without experimental processing. Proved by the simulation at the local range and global range, R = 408.726, r = 406.855, t_0_ = 0.712, h = 365.505, and a = 54.415 is the best solution in the range of structure parameters that Table 2 actually shows.

This study provided an optimization formula for micro-resonators based on PSO-BP and NSGA-II under the limitations of specific processing conditions, avoiding experimental processing, and can be used in any one of the manufacturing processing. Obviously, it will make a difference in guiding the optimization of MEMS hemispherical resonator gyroscopes. 

The structure parameters that mainly affect the performance parameters of the hemispherical shell resonator are obtained by analyzing the thermoelastic damping losses. The finite element simulations with variables controlling are used to obtain a dataset of the performance parameters of the hemispherical resonator when the structure parameters vary. By optimizing the weight, deviation, and number of hidden layer neurons via PSO, as Section 3 claims, the problem of the BP neural network that falls into a locally optimal solution is easily resolved. Finally, the limitations of molding defects under the process conditions are considered constraints, and the optimized BP neural network is used as the objective function to obtain the best structure parameters.

At the end of Section 3, validated by simulation, the result of the simulation is almost similar to the result of NSGA-II output; we prove that this approach to optimize the resonator is available. It solves the problem that it is difficult to design the best structure under a specific process because the structure parameters are under the limitation of process conditions. 

However, there are also some limits in this study. First, the weight of the Q factor and frequency difference is not clear, although the Pareto frontier gives the non-dominated optimal solution set, to what an extent that Q factor and frequency difference occupies the comprehensive performance should be quantized clearly; another limit is that what we study is in a selected numerical interval, in different numerical intervals, there might be a great difference among the mappings, so, the numerical intervals selected in an optimization should not be too large.

Moreover, there would be a sudden change when the hemispherical resonator changes from a standard one to a nonstandard one because that the sudden change in the structure results in a sudden change in the path length for the irreversible heat flow.

For the next work, the characteristic frequency of the operating mode can be limited under 10 kHz or 15 kHz as it is a little high for the frequency of 31 kHz from this study; another job is to discover the particular cause of the sudden change when the hemispherical resonator changes from a standard one to a nonstandard one.

## Figures and Tables

**Table 1:** Characteristics of different manufacturing processes.

Manufacturing Process	Structure	Limits
Glass blow molding	Large birdbath with thin bottom	Complex process
Laser ablation	Relatively standard hemisphere	Complex assembly, Difficult to machine complex surface
Thin film deposition	Hemisphere with non-standard aspect ratio	Large surface roughness, Large frequency cracking

**Table 2:** Varying range of structure parameters.

R (μm)	r (μm)	H (μm)	h (μm)	A (μm)
301~502	299~500	251~552	249~550	50~100

**Table 3:** Results of variable-controlling simulation.

R (μm)	r (μm)	H (μm)	h (μm)	a (μm)	Characteristic Frequency	Q Factor	Near Frequency
301	300	301	299.5	75	52,490.33	40,665.81	61,882.17
451	450	451	450	90	16,207.31	58,436.62	26,140.7
401	400	401	400	79	18,720.86	59,591.18	14,057.15
381.25	380	381	380	75	21,309.55	78,224.75	24,546.6
501	500	501	500	87	11,118.6	74,553.32	10,232.95

**Table 4:** The combinations of structure parameters around point 12 at the local range.

Difference in R (μm)	Difference in r (μm)	Difference in H (μm)	Difference in h (μm)	Difference in a (μm)	Characteristic Frequency	Q Factor	Frequency Difference
−0.05	0	0	0	0	31,537.99	41,554.92	6556.38
0.07	0	0	0	0	30,954.05	43,323.97	10,578.55
0	−0.09	0	0	0	30,899.5	43,650.8	11,220.80
0	0.06	0	0	0	31,857.08	41,908.96	7877.25
0	0	0	−0.08	0	30,470.41	42,853.35	8775.74
0	0	0	0.04	0	30,857.08	42,059.67	8877.25
0	0	−0.5	−0.5	0	31,246.31	42,265.57	8232.49
0	0	2.5	2.5	0	31,357.19	42,211.96	8381.35
0	0	0	0	−1.2	30,588.4	42,335.84	8910.82
0	0	0	0	2	32,744.45	42,225.87	6780.15

**Table 5:** The combinations of structure parameters around point 12 at the global range.

Difference in R (μm)	Difference in r (μm)	Difference in H (μm)	Difference in h (μm)	Difference in a (μm)	Characteristic Frequency	Q Factor	Frequency Difference
−0.5	0	0	0	0	28,689.24	36,126.38	8078.58
0.2	0	0	0	0	30,569.92	45,706.89	14,725.43
0	−0.4	0	0	0	30,490.75	50,160.07	20,698.64
0	0.3	0	0	0	29,136.87	38,791.72	4486.53
0	0	0	−0.35	0	27,749.8	46,513.92	10,631.14
0	0	0	0.4	0	35,183.68	41,293.17	5850.65
0	0	−20	−20	0	30,533.86	42,578.94	7373.49
0	0	32	32	0	32,501.35	41,883.58	10,083.70
0	0	0	0	−18	21,794.66	33,747.67	17,578.09
0	0	0	0	7	36,686.79	41,510.09	2971.12

**Table 6:** The comparison between the simulation results of this study and experimental data from the relevant literature.

Radius of Shell (μm)	Thickness of Shell (μm)	Depth of Shell (μm)	Radius of Anchor (μm)	Characteristic Frequency of Operating Mode (Hz)	Frequency of Spurious Mode (Hz)	Q Factor	Remark
400	1.8	—	—	28,012.21	—	14,365	[7]
650	1.7	160	—	13,649	—	22,000	[19]
408.73	1.87	365.51	54.42	31,016.25	39,555.27	42,454	This study

## Data Availability

Not applicable.
